# Effects of Polysaccharide Coating on Cell-Surface Association and Endocytic Uptake of PLGA Nanomicelles in MCF-7 Cells

**DOI:** 10.3390/pharmaceutics18010017

**Published:** 2025-12-22

**Authors:** Abdulkadir Bahadir Alkan, Esma Nur Develi Arslanhan, Fatemeh Bahadori, Muhammed Zahid Kasapoglu, Fahri Akbas, Seda Susgun, Zahra Eskandari, Ebru Toksoy Oner

**Affiliations:** 1 Department of Biotechnology, Institute of Health Sciences, Bezmialem Vakif University, Fatih, 34093 Istanbul, Turkey; bahadiralkan.123@gmail.com (A.B.A.); esmadeveli595@gmail.com (E.N.D.A.); 2 Department of Analytical Chemistry, Faculty of Pharmacy, Istanbul University-Cerrahpasa, Buyukcekmece Campus, 34500 Istanbul, Turkey; 3 Department of Pharmaceutical Biotechnology, Faculty of Pharmacy, Bezmialem Vakif University, Fatih, 34093 Istanbul, Turkey; 4 Institute of Nanotechnology and Biotechnology, Istanbul University-Cerrahpasa, 34500 Istanbul, Turkey; muhammedkasapoglu@entertech.com.tr; 5 Department of Medical Biology, Faculty of Medicine, Bezmialem Vakif University, Fatih, 34093 Istanbul, Turkey; fahriakbas@gmail.com (F.A.); sedasusgun@gmail.com (S.S.); 6 IBSB-Industrial Biotechnology and Systems Biology Research Group, Department of Bioengineering, Marmara University, 34722 Istanbul, Turkey; zeskandari@mgh.harvard.edu (Z.E.); ebru.toksoy@marmara.edu.tr (E.T.O.)

**Keywords:** cellulose, endocytosis, levan, MCF-7, nanoparticle, PLGA

## Abstract

**Background:** Targeting cancer tumors using PLGA (Poly(D, L-lactide-co-glycolide)) nanoparticles (NPs) requires clathrin-mediated endocytosis (CME) and lysosomal degradation to provide release within cancer cells. However, Caveolae-mediated endocytosis (CavME) provides lysosomal escape, which is favorable in oral applications. Macropinocytosis (MPC) is a non-targeted way of endocytosis, used by immune cells. **Methods:** In this proof-of-concept study, we investigated how polysaccharide surface coatings modulate the endocytic uptake of FITC-labeled PLGA nanomicelles (FPM) in MCF-7 breast cancer cells using spectrophotometry. This research involved the surface modification of FPM using polysaccharides: cellulose (FPCM) as a polyglucan and Halomonas Levan (FPLM) as a polyfructan, to modify the NP and cell-surface association. **Results:** MPC was found to be the major internalization pathway for the nanomicelles ~200 nm. However, after surface modification, FPCM and FPM remained highly MPC-dependent with additional CavME/CME involvement, whereas FPLM showed relatively reduced MPC dependence and a higher CME contribution. **Conclusion:** Overall, the results indicate that simple polysaccharide coatings can bias the relative use of MPC, CME, and CavME for PLGA nanomicelles in MCF-7 cells, providing a basis for pathway-oriented nanocarrier design. Validation by flow cytometry, studies in additional breast cancer cell lines, and transporter-level investigations will be needed to generalize and refine these findings.

## 1. Introduction

NanoDrug Delivery Systems (NDDS) are particles consisting of natural or synthetic materials designed to deliver drug molecules, achieving higher activity and a better toxicity profile with lower doses than traditional pharmaceutical forms [1]. Targeting cancer tumors using the Enhanced Permeability and Retention (EPR) effect is one of the most studied properties of NDDS. This so-called “passive targeting strategy” is based on the size of the NDDS, which needs to be smaller than the enhanced gap between the endothelial cells at the cancer tumor site and bigger than the gap between these cells in healthy tissue to provide accumulation of NDDS only at the cancer tumor area [2]. Although this is a very effective way of targeting cancer, the lack of a lymphatic system in this area causes extremely high fluid pressure, which leads to the repletion of accumulated NDDS. High interstitial fluid pressure is the most important obstacle in targeted cancer therapy, especially when it causes the release of encapsulated chemotherapy agents from NDDS in the extracellular matrix and consequently results in drug dispersion in the bloodstream [3].

Active targeting aims to exploit receptors that are overexpressed on cancer cells to facilitate the cellular internalization of NDDS and to reduce their nonspecific distribution in the bloodstream. Various active targeting strategies have been developed to complement passive targeting and to enhance intracellular drug delivery. One such approach is to present glucose units on the surface of NDDS because tumor cells require more nutrients than healthy cells [4,5].

The ability of NDDS to present a sufficient amount of glucose units to membrane receptors has long been a concern. Small glucose molecules conjugated to short polymer chains can remain shielded within the hydrodynamic volume of the nanoparticles, whereas long polymer chains that successfully expose glucose to the cell surface may also decrease the internalization rate due to steric hindrance [6]. As an alternative, NDDS can be surface-modified with polymers composed of poly-hexose or poly-pentose groups (polysaccharides). Previous studies have suggested that polysaccharides made of glucose derivatives, such as chitosan, can promote preferential association with cancer cells in a manner reminiscent of glucose-based targeting [7]. Nonetheless, these effects are likely to involve a combination of physicochemical changes and multiple carbohydrate–receptor interactions, and the precise receptor-level mechanisms remain to be elucidated in future work.

The pathway involved in the cell internalization is of huge importance in terms of the anticancer efficacy of NDDS. Nanoparticles (NP) internalized via clathrin-mediated endocytosis (CME) become the targets for the lysosomal compartments for degradation, whereas caveolae-mediated endocytosis (CavME) escapes these compartments [8,9]. Thus, CavME is favored when plasmids, nucleotides, and genes are being carried by the NDDS in order to protect the payload from degradation within lysosomes, and CME is preferred in targeting cytotoxic small molecules such as chemotherapeutic molecules to provide their release by endosomolysis [8]. CavME is also desired in oral drug delivery, where NDDS needs to penetrate the intestinal membrane to escape lysosomal degradation [10].

Poly (D, L-lactide-co-glycolide) (PLGA) is a copolymer widely used in the preparation of NDDS for application via many routes, including oral, intraperitoneal, and intravenous, due to its convenience in terms of bioavailability, biocompatibility, and successful drug delivery history. A previous study reported that the cell internalization of PLGA in Caco-2 gut epithelial cells is less than in other tissues [11]. This could be considered a disadvantage in using PLGA nanomicelles (PNM) as oral drug delivery systems.

On the other hand, some studies have modified the surface of PNM using peptides or aptamers to study or enhance the cell internalization pathway of PLGA NPs [12,13]. These studies have used chemical conjugation as a method for the incorporation of PLGA with cell-internalization mediators. However, chemical reactions could create toxic artifacts or increase the toxicity of PLGA. Thus, incorporating PLGA with the cell-internalization mediators via physical interaction would be a safer strategy for studying and modifying the cell internalization of this NP. Furthermore, to track the entry of NPs into the cell, it may not be the right approach to follow the intracellular entry of the drug molecule carried by the NP, because there is a possibility of cell internalization of the released drug while the NP is left in the outer cell environment. Therefore, it would be useful to track the entry of the nanoparticle itself into the cell, not the carried drug molecules.

In this study, fluorescein isothiocyanate (FITC) labeled self-assembled PNM were prepared and physically incorporated with the long-chain polysaccharides, including cellulose (C) as a poly-glucan and levan as a poly-fructan. Levans, including Halomonas levan (HL) produced by bacteria such as *Halomonas smyrnensis*, are biocompatible polymers [14] with linear β-(2–6) and partly branched β-(2–1) glycosidic linkages [15,16]. Thus, the effect of poly-glucans and poly-fructans on the alternation of the cell internalization pathway of PNM was investigated compared to that of bare PLGA, while FITC labeling provided the possibility of tracking the cell internalization pathway using a fluorescence microscope. It was hypothesized that surface modification of PLGA NPs by the polysaccharides with polymeric structure will expose a larger amount of sugar units to the cancer cells, resulting in better enhancement of the cell internalization. The uptake mechanisms of bare and surface-modified PNM were elucidated by using the specific pharmacological inhibitors, including genistein (CavME inhibitor), dynasore (dynamin inhibitor), 5-(N-ethyl-N-isopropyl) amiloride (EIPA, macropinocytosis (MPC) inhibitor), chlorpromazine (CME inhibitor), methyl-β-cyclodextrin ((MβCD) cholesterol depletion agent), all of which have previously been reported in the literature [10].

It is noteworthy that several studies have reported that carbohydrate-decorated NDDS can achieve facilitated cellular internalization via nutrient transporters such as GLUT1 or GLUT5, resulting in higher efficacy than their non-decorated counterparts [5]. Although GLUT transporters play a key role in the uptake of carbohydrate derivatives, this pathway was not specifically examined in the present work. Whether the surface-modified nanocarriers developed here interact with GLUT receptors remains an open question and should be addressed in future studies.

Hormone receptor–positive, luminal breast cancer remains the most prevalent molecular subtype worldwide, and MCF-7 cells are a well-established in vitro model for this estrogen-dependent phenotype. For this reason, MCF-7 cells were selected in the present work to obtain a first mechanistic insight into how polysaccharide-coated PLGA nanomicelles are internalized by hormone-dependent breast cancer cells. The findings are therefore most directly relevant to luminal, hormone-driven tumors, and cell internalization profiles may differ in hormone-independent or more aggressive subtypes such as triple-negative breast cancers [17,18,19].

## 2. Materials and Methods

### 2.1. Materials

Pharmaceutical inhibitors namely genistein, dynasore, 5-(N-ethyl-N-isopropyl) amiloride (EIPA), chlorpromazine, MβCD, also, PLGA (lactide:glycolide (50:50), mol wt. 30,000–60,000), FITC labeled PLGA (PLGA-Fluorescein/PLGA-FITC, mol wt. 10,000–20,000), Tween 80 (suitable for cell culture) Methyl cellulose, Sulforhodamine B (SRB) sodium salt, glacial acetic acid, trichloro acetic acid, HPLC grade acetone, Phosphate Buffer Saline (1 × PBS) and Trizma^®^ base were purchased from Sigma-Aldrich (St. Louis, MO, USA). BD Cell Lysis Buffer was obtained from Fisher Scientific (Pittsburgh, PA, USA). Methylcellulose, viscosity: 15 cP, BioReagent, suitable for cell culture, was also obtained from Sigma-Aldrich.

### 2.2. Methods

#### 2.2.1. Natural Synthesis of Halomonas Levan (HL)

*Halomonas* Levan was produced via microbial fermentation under controlled bioreactor conditions. The polymer was recovered from the medium and further purified by Anion-Exchange Chromatography as reported previously [20].

#### 2.2.2. Fabrication of Nanomicelles of PLGA (PM), FITC Labelled PLGA (FPM), PLGA-Levan (FPLM), and PLGA-Cellulose (FPCM)

In all experiments, FPM was compared with PM to eliminate any possible toxicity caused by the building blocks of PLGA.

The PM was prepared using the o/w emulsification-evaporation method. 20 mg PLGA was dissolved in 1.25 mL of acetone and dropped on d.d water containing %0.01 Tween 80. The organic phase was evaporated at RT on a magnetic stirrer [21].

FPM was prepared using the method mentioned above by using 1 mg PLGA-FITC and 19 mg PLGA co-dissolved in the same amount of acetone. FPLM and FPCM were prepared by adding optimized amounts of HL and C to the aqueous phase. The quantities of HL and C were optimized to obtain mono-dispersed PLM and PCM with sizes less than 300 nm. For this purpose, 20 and 7.5 mg of HL and C were added, respectively, to the 0.01% Tween 80 containing d.d. water, while the organic solvent comprised 1 mg PLGA-FITC and 19 mg PLGA [21].

#### 2.2.3. Characterization of NPs

The size, shape, and surface charge of PM, FPM, FPLM, and FPCM were determined using the Dynamic Light Scattering (DLS) method (Malvern Instruments Ltd., Malvern, UK). The dispersant was chosen as water, and the measurement angle was set as 173 with the refractive index of 1.59 and absorption of 0.010. The surface zeta potential of particles was determined by the laser Doppler micro-electrophoresis method (Malvern Instruments Ltd.). All the measurements were repeated 3 times at RT. Particle size was reported as number, volume, and intensity distribution to ensure monodispersity of all particles.

The interaction of PLGA and polysaccharides in the structure of nanomicelles was determined using Differential Scanning Chalorimetry (DSC). In this method, any physical change in the structure of NP caused by absorption or the release of heat is associated with a recorded thermogram. Thus, the position of HL or C in the structure of PNM was determined using DSC to ensure the incorporation of C and HL with PLGA on the shell, not in the core. Measurements were performed using a Perkin Elmer Jade type DSC instrument (Shelton, CT, USA) between 0 and 260 °C at 10 °C/min heating rate under dynamic argon. The results were evaluated by the Pyris software (PerkinElmer, Inc., USA; available at: https://www.perkinelmer.com/) [22,23]. The crude HL and C were used equivalent to their amounts used in NP synthesis, while FPM, FPLM, and FPCM were used as synthesized NPs after lyophilization.

#### 2.2.4. Cell Culture

MCF-7 (HTB-22™) obtained from ATCC was used to study the cell internalization pathways of PM, FPM, FPLM, and FPCM. The cells were cultured in DMEM-F12 medium containing 10% fetal bovine serum (FBS) (Sigma-Aldrich, USA) and 1% penicillin-streptomycin (P/S) (Sigma-Aldrich, USA) solution (100 U/mL penicillin and 100 μg/mL streptomycin). Cells were grown in a 5% CO_2_ and 95% humidified incubator at 37 °C.

The cells were cultured at a concentration of 4 × 10^4^ cells/cm^2^ in 96 and 48 wells for performing the cytotoxicity and cell internalization assays, respectively. The prepared formulations were applied directly to the cell culture on the fresh media in a 10% ratio (*v*/*v*) without agitation. All nanomicelle dispersions were freshly prepared and used within 24 h. The size distributions were monomodal with PDI values < 0.21, consistent with colloidally stable PLGA nanoparticles under the experimental conditions.

#### 2.2.5. Toxicity Assays

The toxicity of NPs was studied to determine each NP’s non-toxic dose. PM, FPM, FPLM, and FPCM and their diluted forms in d.d. water were prepared. The dilutions of nanoformulations were prepared by decreasing the concentration by 50% in four consecutive solutions. Measurements were done in 6 and 24 h (*n* = 9).

Since the inhibitors of cell internalization pathways are also toxic to living cells [10] the toxic dose of each pharmacological inhibitor was determined on MCF-7 cells. The toxicity of pharmacological inhibitors was examined in five consecutive concentrations prepared according to previous literature, considering their toxicity on similar cell lines [10] in 24 h. Dynasore, genistein, and EIPA were dissolved in 0.01% DMSO, while chlorpromazine and cyclodextrin were dissolved in 1 × PBS. The concentration of each inhibitor that results in obtaining 100% cell viability was accepted as the non-toxic dose.

The cell survivor was studied using the sulforhodamine B (SRB) cell viability assay. Briefly, after the application of NPs or inhibitors, and incubating the cells for the mentioned periods, the media was removed, and the cells were fixed using 20% (*w*/*v*) cold trichloroacetic acid (TCA) followed by further incubation of cells in 4 °C. After removing TCA, each well was washed 3 times with cold water, dried, and stained with 100 µL/well of 0.4% (*w*/*v*) SRB in 1% acetic acid (AA) and incubated for 30 min at room temperature. Unbounded dye was washed with 1% AA, and the bound dye was solubilized using 200 µL/well Tris base (10mm) at pH 10. Optical density was measured at 515 nm using a microplate reader (Varioskan™, Thermo Scientific, Waltham, MA, USA) [24].

#### 2.2.6. Determination of the Cell Internalization Period of NPs

In earlier studies, the effects of internalization inhibitors on the uptake of the surface-modified PLGA NPS were carried out at very different intervals from 2 to 24 h. Thus, determining the minimum period needed to internalize FP, FPLM, and FPCM into the cancer cells was crucial to investigating the inhibitory effect at the right time ranges. For this purpose, PM, FPM, FPLM, and FPCM were applied to the cells in 48-well plates, and the experiment was ended at the 1st, 4th, 6th, and 24th hours by collecting the media, washing the cells with 1 × PBS, and adding the cell lysis buffer. The cell lysates containing internalized fluorescent PLGA NPs were then collected and centrifuged, and the emission of FITC was measured at λ_em_ = 528 nm with λ_ex_ = 485 nm using the ELISA reader instrument (Synergy-H1, BioTek Instruments, Inc., Winooski, VT, USA). The results were reported as Relative Fluorescence Units (RFU). The PM (without incorporation with FITC) and the non-treated cells were used as controls.

#### 2.2.7. Cell Internalization Mechanism of NPs

After determining the optimized period for obtaining the maximum ratio of endocytosis, the cell internalization pathway of NPs was defined using the pharmaceutical inhibitors consisting of Dynasore (dynamin inhibitor), genistein (CavME inhibitor), EIPA (MPC inhibitor), chlorpromazine (CME inhibitor), and cyclodextrin (cholesterol depletion agent) using a modified version of the previously reported method [10]. Basically, each internalization pathway was inhibited using the related specific inhibitor by adding the non-toxic doses of each inhibitor to the cells in 48-well plates and waiting for 30 min. This was followed by adding the non-toxic amounts of NPs to the cells (without removing the media or inhibitors) and incubation for 6 h (the optimized period needed for obtaining the max. ratio of cell internalization). The emission of fluorescent PLGA NPs accumulated inside the cell was measured using an ELISA reader instrument (and reported as RFU) after obtaining the cell lysates as mentioned above. The images of cells were recorded under the Zeiss Observer Z1 fluorescent microscope (Carl Zeiss Microscopy GmbH, Jena, Germany). without collecting the media.

In all experiments, non-treated cells were used as controls, and the cells treated with regular PM (non-labeled with FITC) were used to ensure the obtained fluorescent emission was not due to the turbidity of the samples caused by the degraded polymeric material.

#### 2.2.8. TEM Analysis

Transmission electron micrographs were obtained using a JEOL JEM-2100 microscope (JEOL Ltd., Tokyo, Japan) operated at 80 kV. For sample preparation, copper grids (0.037 mm) were loaded with PM, FPM, FPLM, or FPCM dispersions at a total nanoparticle concentration of 1 mg/mL. Negative staining was achieved by applying a 2% (*w*/*v*) aqueous uranyl acetate solution (Electron Microscopy Sciences, Hatfield, PA, USA). Excess stain was removed with double-distilled water, and the grids were air-dried at room temperature before imaging.

#### 2.2.9. Statistical Analysis

The values for cell internalization of FPLM, FPCM, FPM, and PM with inhibitors, compared to non-inhibited NPs, were analyzed using one-way ANOVA. To provide significance within the groups, a post-hoc Tukey’s HSD test was performed to investigate differences between groups. Statistical significance is reported at a level of 0.05.

## 3. Results

### 3.1. Particle Size Distribution and Zeta Potential of NPs

Table 1 shows the particle size distribution of PM, FPM, FPLM, and FPCM. The DLS and zeta graphs are also presented in Supplementary Data S1.

As shown in Table 1, all particles were obtained as monodispersed NPs with significantly low PDI values. The sizes of all NPs were in a good range for targeting cancer therapy, and the surfaces of NPs were found to be all negatively charged and in a narrow range as well.

### 3.2. Determination of Incorporation of PLGA with Polysaccharides Using DSC

**Figure 1:**
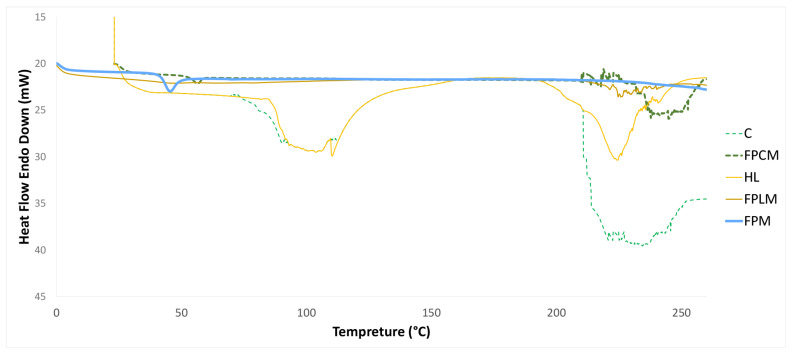
DSC thermogram of HL, C, FPM, FPLM, and FPCM.

Figure 1 shows the results of the DSC assay of HL, C, FPM, FPLM, and FPCM.

**Scheme 1:**
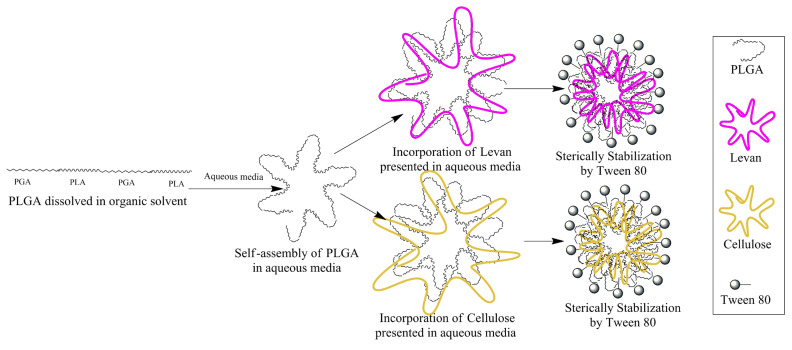
The schematic view of the preparation method for FPLM and FPCM, and the position of each component in the structure of the final NP.

Our group previously reported on the DSC thermogram of NPs made from HL and PLGA incorporation [21]. The results obtained in this study are in complete agreement with our previous studies, where the initial endothermic peak around 100 °C was attributed to the elimination of free and bound water. In contrast, the second endothermic peak at 225 °C was caused by thermal decomposition. HL exhibits an amorphous structure and does not show melting behavior [21]. The same endothermic peaks are observed in the DSC thermogram of C at 100 and 220–240 °C, indicating dehydration of surface water and decomposition, respectively. These findings are consistent with earlier results [25,26]. In the DSC curve of PLGA, a minor endothermic peak at 56.0 °C, corresponding to the glass transition temperature (Tg), was observed. Still, no melting behavior was detected within the operational range of the DSC. The incorporation of FP with HL and C to produce FPLM and FPCM caused a significant decrease in the enthalpy of the HL and C decomposition peak [21]. The intensity of the Tg peak of FP was also reduced in both FPLM and FPCM. These data revealed the presence of the HL and C in the outer shell of produced nanomicelles. In our previous study [21], we also conducted FT-IR studies that clearly showed the incorporation of the interaction between the negatively charged oxygens of the C=O groups in PLGA with the positively charged hydrogens of O-H groups in Levan. Thus, the overall incorporation of HL and C can be illustrated as Scheme 1.

### 3.3. Toxicity Assays

The toxicity of NPs and the pharmacological inhibitors were defined using the SRB method by preparing five doses of each pharmacological inhibitor below and above their previously reported non-toxic concentrations [10]. The dose that resulted in 100% cell viability was designated as the non-toxic dose. The determined concentrations were further used to determine the cell internalization pathways. Table 2 shows the non-toxic doses of each inhibitor on the MCF-7 cell lines.

**Figure 2:**
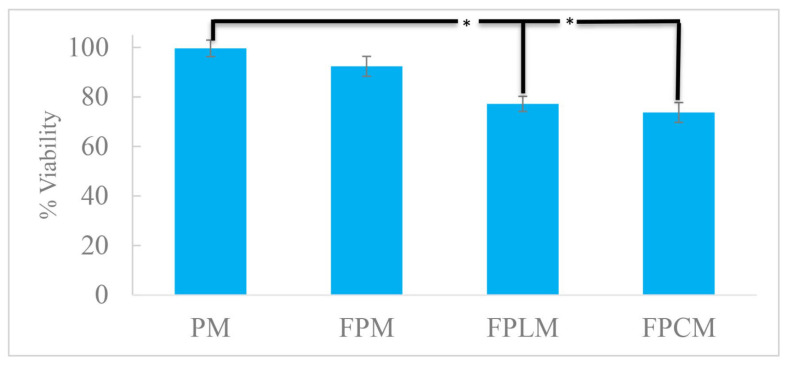
Cell viability of the MCF-7 cells exposed to the fabricated NPs for 24 h, showing the minor toxicity of FPLM and FPCM (*n* = 3, *—*p* < 0.05).

The toxicity of NPs was also studied using the SRB method on the MCF-7 human breast cancer cell lines. Figure 2 shows the toxicity of PN, FPM, FPLM, and FPCM. Introducing FITC to the structure of PM caused a minor increase in toxicity, which was further increased by surface modification by HL and C. All nanoparticle formulations preserved MCF-7 viability above 70%. Compared with untreated cells, only FPLM and FPCM induced a modest but statistically significant decrease in viability (one-way ANOVA, Tukey’s test, *p* < 0.05), whereas FPM did not differ significantly from the control. Thus, the produced NPs were used in cell internalization studies without further dilutions.

### 3.4. Determination of Max. Cell Internalization Period of NPs

**Figure 3:**
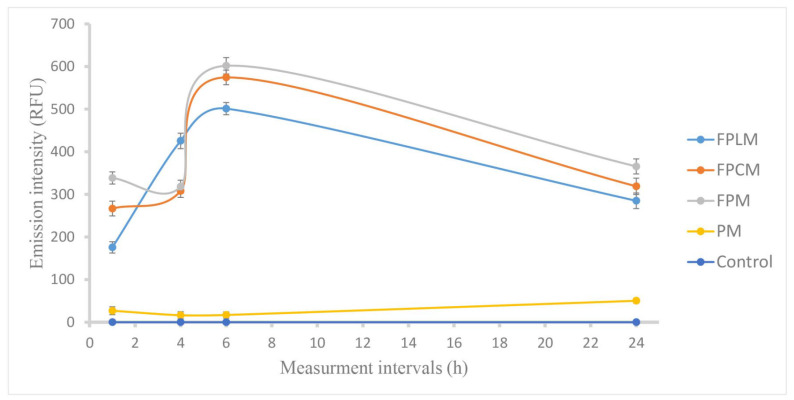
Cell internalization of FPM, FPSM, and FPLM to the MCF-7 cells by time, based on the fluorescence intensity of FITC conjugated to PLGA reported as RFU values, showing the maximum internalization of NPs at the end of 6 h. PM and non-treated cells (control) were used to measure solely the fluorescence of FITC (*n* = 3).

To examine the effects of pharmacological inhibitors at optimal times, the duration required for maximum cell internalization was investigated. Figure 3 shows the cell internalization values based on the emission intensity of FITC. PM and control were used to ensure no fluorescent interference was caused by the polymeric material or by the process.

The results showed that maximum cell internalization is obtained within 6 h, and a decrease in fluorescence intensity is observed at 24th h, probably due to the FITC degradation. Based on these results, 6 h was chosen as the optimum period for the cell internalization studies.

### 3.5. Cell Internalization Mechanism of NPs

The non-toxic doses of pharmaceutical inhibitors (as reported in Table 2) and the non-toxic concentrations of the FITC-labeled NPs were used to determine the cell internalization mechanisms. Based on the optimization studies, all assays were finalized at the end of 6 h of incubation. The nonlabelled PM was also used as a control. The cell internalization inhibition of each inhibitor for each NP was compared to the cell internalization of NPs without any inhibitor. Furthermore, intragroup comparisons were used to identify the NP that each inhibitor inhibited most efficiently and the NP that was most effectively inhibited by all inhibitors.

**Figure 4:**
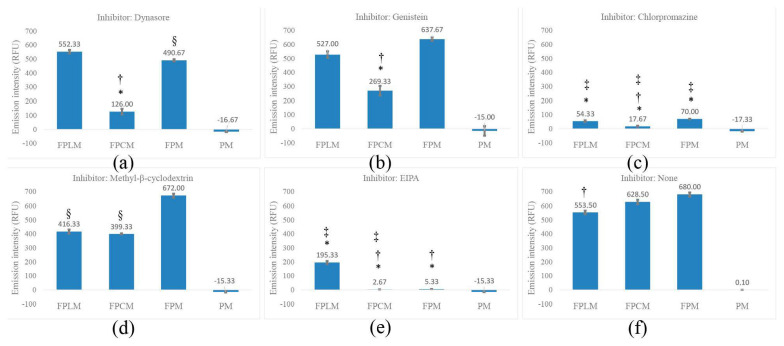
Cell internalization ratios of FPLM, FPCM, FPM, and PM on MCF-7 cell lines with (**a**) dynasore, (**b**) genistein, (**c**) chlorpromazine, (**d**) MβCD, (**e**) EIPA, and (**f**) no inhibitor. Statistical significance is shown by *—*p* < 0.001, more inhibited compared to the internalization of the same NP without inhibitor, §—*p* < 0.05 more inhibited compared to the same NP without inhibitor, †—*p* < 0.001 the most inhibited NP within the same inhibitor group, and ‡—*p* < 0.001 most effective inhibitor against the same NP group. The PM was used as the negative control in all assays (n = 3).

For all formulations, inhibition of macropinocytosis (MPC) with EIPA (Figure 4e) significantly reduced cellular uptake compared with the non-inhibited control (Figure 4f), indicating that MPC is a major route of entry for these ~200 nm particles. The strongest relative inhibition by EIPA was observed for FPM and FPCM (Figure 4e, *p* < 0.001), supporting macropinocytosis as the primary uptake pathway for these two formulations.

In contrast, for HL-modified FPLM, inhibition of clathrin-mediated endocytosis (CME) with chlorpromazine (Figure 4c, *p* < 0.001) produced the largest reduction in uptake, whereas EIPA had a more moderate, although still significant, effect. Based on these data, CME is classified as the primary pathway and MPC as a secondary pathway for FPLM, with caveolae-mediated endocytosis (CavME; genistein and MβCD) contributing to a lesser extent (Figure 4b,d, *p* < 0.001).

Overall, the inhibitor profile suggests that macropinocytosis is an important route for all three polysaccharide-coated PLGA nanomicelles, but polysaccharide composition shifts the balance between MPC, CME, and CavME. FPM and FPCM are predominantly internalized via MPC, while FPLM shows a stronger dependence on CME with additional contributions from MPC. The detailed inhibition percentages and *p*-values used to define primary and secondary pathways are summarized in Supplementary Data S3.

**Figure 5:**
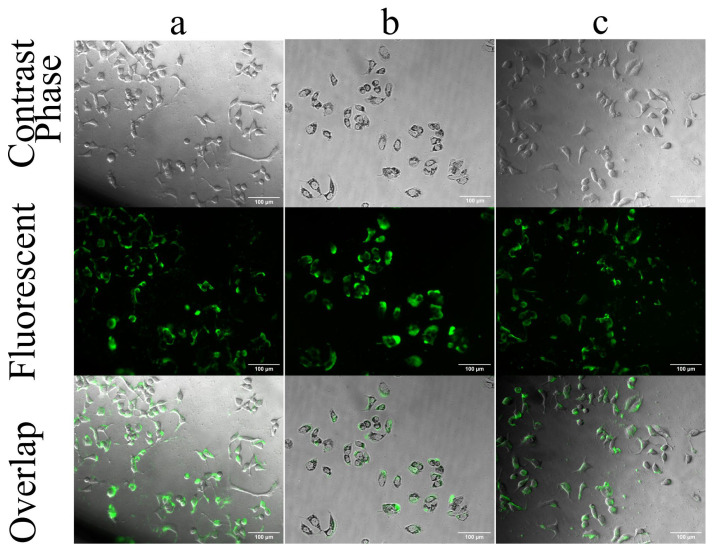
Selected fluorescent microscope images of MCF-7 cells treated with FPCM. (**a**) cells treated with FPCM for 6 h without inhibitor, (**b**) cells treated with chlorpromazine and FPCM, and (**c**) cells treated with MβCD and FPCM. The overlapped panel shows the accumulation of fluorescent dye inside the cell, while the middle fluorescent panel shows both the dyes inside and outside of the cells. Images related to the results of other inhibitors are presented in Supplementary Data S2. The statistical analysis of cell internalization is presented in Figure 4, and images were acquired at 20× magnification. The scale bar shows 100 μm.

Figure 5 shows the fluorescent microscope images of some selected test groups. Figures of all groups are presented as Supplementary Data S2.

Figure 5a shows the internalization of un-modified FPLM. The FITC emission recorded by the fluorescent micrograph is well-accordant with that of the overlapped image showing the internalization of almost all fluorescent particles into the MCF-7 cells. The images of the cells treated with NPs and inhibitors were captured before collecting the media to avoid cell death and the potential for misleading results. In Figure 5b,c, the inhibition of internalization of FPCM by chlorpromazine and MβCD is shown. Although the fluorescent images show high FITC emission, the overlapped images show that only a part of FITC is internalized into the cells. The accumulation of the fluorescence dye in the middle panel is due to the accumulation of FITC-labeled PNM in the outer environment of cells, while inhibitors prevent their internalization. The images of the internalization of FPCM associated with other inhibitors are presented as Supplementary Data S2 [12]. Figure 5 is combined with Supplementary Data S2 to provide a better comparison.

### 3.6. TEM Images of NDDS

**Figure 6:**
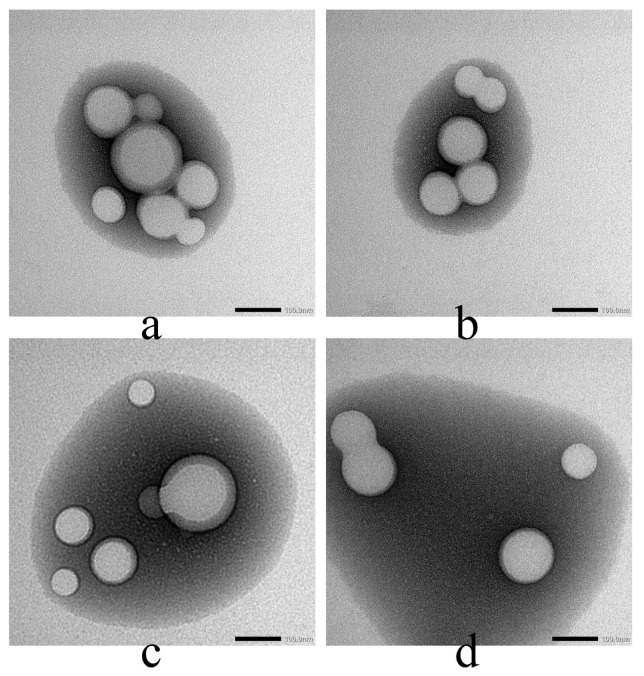
The TEM images of (**a**) PM, (**b**) FPM, (**c**) FPLM, and (**d**) FPCM, Image acquired at 40,000× magnification; scale bar = 100 nm.

Figure 6 shows the TEM images of all synthesized particles. All particles show sizes less than 200 nm, which is much smaller than the obtained results in the DLS studies. This shows the increase in hydrodynamic particle size in aqueous media. The morphology of particles did not show a significant change after surface modification.

## 4. Discussion

The size measurements of PNM showed that the incorporation of 20 mg PLGA with 20 and 7.5 mg of HL and C, respectively, resulted in obtaining monodisperse spherical NPs (Supplementary Data S1) with appropriate sizes (Table 1) to benefit from the EPR effect and targeted cancer therapy [2]. The incorporation of HL with PLGA was previously discussed in detail by our group [21,24]. The number distribution of the size of FPLM, observed at 120.8 nm, was consistent with our previous results. To the best of our knowledge, this is the first report on the incorporation of C with PLGA. FPCM also showed a particle size distribution of 209.3 nm (Table 1). The sizes of FPLM and FPCM were not significantly different than those of PM and FPM. However, FPM was slightly larger than PM. Since the particle sizes were measured using DLS, where the hydrodynamic size is calculated [27], the higher particle size of FPM could be attributed to the localization of FITC at the outer shell of PNM and the accumulation of ionized water molecules around FPM. For long-term storage, the nanomicelles are intended to be converted into lyophilized powders and re-dispersed immediately before use, a common strategy to further preserve colloidal stability of PLGA-based systems [23].

Previously, FITC was used as a surface modifier to obtain negatively charged NPs [28]. This hypothesis is supported by the higher negative charge of FPM compared to PM (Table 1). The incorporation of HL and C with FPM did not cause a significant change in its surface charge, and compiling, the variations in the size and surface charge of FPLM and FPCM were not significant enough to alter the cell internalization pathways of these particles [10,29]. Thus, it can be concluded that the variations in cell internalization pathways were solely due to the different inhibitors.

DSC results revealed the localization of C and HL at the outer shell of FPCM and FPLM, respectively. In terms of modifying the cell entry pathway, keeping C and HL in the outer shell of the PNM was very important to achieve the aim of this research. The encapsulation of C and HL by PLGA during NP synthesis would only involve the cell membrane with the PLGA polymer. DSC studies showed a significant decrease in the thermogram of PLGA upon incorporation with HL and C. Complete disappearance of the endothermic peak of PLGA at 56.0 °C in the thermogram of FPLM and its significant decrease in that of FPCM prove the settlement of PLGA at the inner part of FPLM and FPCM. Furthermore, the endothermic peaks related to FPCM and FPLM at 220–240 °C were predominated by C and HL. These findings are consistent with our earlier reports, where we thoroughly discussed the incorporation of HL with PLGA [21].

To investigate the internalization mechanism of the synthesized NPs with an adequate cell population, it was crucial to establish the non-toxic concentrations of both the pharmacological inhibitors and the produced NPs. Although FPCM and FPLM showed higher toxicity than FPM (Figure 2), this toxicity was not significant to hinder further experiments. Therefore, the optimal time required to achieve maximum internalization of the NPs was determined using the highest concentrations of FPLM and FPCM, which were 40 mg and 27.7 mg, respectively. As it is shown in Figure 3, the optimal period for obtaining maximum cell internalization for all NPs was determined as 6 h. This result was in agreement with previous studies that showed the maximum accumulation of PNM on the cell organelles within 4–24 h [11]. FPM was the NP with the highest cell internalization ratio, followed by FPCM and FPLM. This behavior of NPs was observed in the cell internalization mechanism experiments as well.

Chemical inhibition studies suggest that MPC is the predominant internalization mechanism for all formulations, which is consistent with their hydrodynamic sizes. Nevertheless, the higher uptake observed for surface-modified nanoparticles compared with the unmodified formulation, both under control conditions and after partial inhibition, indicates that surface properties modulate the efficiency of this macropinocytic process rather than determining an entirely distinct endocytic route. As shown in Figure 4f, decorating the PNM with HL and C reduced the cell internalization ratio, and this decrease was statistically meaningful only for FPLM (*p* < 0.001). Although FPM was the most internalized NP, it was the least inhibited one. The endocytosis of FPM was most strongly inhibited by EIPA, which targets MPC (Figure 4e), secondly by chlorpromazine, which inhibits CME (Figure 4c) with *p* < 0.001, and Dynasore, which interferes with the dynamin-dependent pathway (Figure 4a) with *p* < 0.05. These results showed that MPC is the major inhibition pathway for the cell internalization of PNM. MPC is a non-receptor mediated, non-selective, and caveolin-independent endocytosis pathway for NPs [30], and it is also used by innate immune cells, like macrophages and immature dendritic cells [31]. This shows the importance of MPC-targeted drug delivery in cancer treatment, gene delivery, and vaccine development.

As shown in Figure 4e, FPM and FPCM were the most inhibited NPs by EIPA, and MPC was the major internalization pathway of these two NPs among all tested pathways. However, the endocytosis of FPLM was less inhibited by EIPA, showing that MPC is not the major internalization pathway of HL-modified PLGA.

Figure 4c shows the inhibition of CME of 3 PLGA-based NPs by chlorpromazine. Although the endocytosis of FPM was significantly inhibited (less inhibited than MPC), the inhibition of FPCM and FPLM was even more severe, with FPCM being the most inhibited. This shows that surface modification of PNM can increase the efficacy of CME compared to MPS in cancer cell internalization (*p* < 0.001). Surface modification of PNM with HL (forming FPLM) slightly shifted the endocytosis pathway of PNM from MPS to CME (*p* < 0.05). CME was found to be the major internalization pathway of FPLM.

CME is highly selective, often driven by the interaction of ligands with specific cell surface receptors. This specificity allows for targeted internalization of molecules or nanoparticles that have been functionalized to bind to these receptors. CME vesicles typically fuse with early endosomes, where the cargo can be sorted for various fates, including recycling, degradation, or transport to specific intracellular locations (e.g., lysosomes, Golgi apparatus). This precise trafficking is advantageous for targeted drug delivery, ensuring that the therapeutic agent reaches its intended destination [32,33,34]. MPC often follows a less well-defined intracellular trafficking route, frequently leading to lysosomal degradation. This can result in the breakdown of therapeutic agents before they reach their intended targets [35]. Within the specific context of cytotoxic drug delivery to MCF-7 breast cancer cells, HL surface modification, which enhances clathrin-mediated endocytosis, appears more suitable than cellulose for promoting CME-driven intracellular uptake. The success of HL in increasing the ratio of CME is due to the success of fructan units in incorporation into the cell membrane. Previously, it has been shown that lipid-based liposomes surface-modified with fructose show almost 3 times higher cell internalization compared to the non-modified ones by interaction with GLUT5 [36]. Our results suggest that fructose may also work separately from GLUT5 to increase cellular uptake.

Dynasore inhibits the act of dynamin (Figure 4a). In CME, clathrin polymerizes to form a coat that facilitates the internalization of NPs. At the same time, dynamin mediates vesicle scission by self-assembling into rings that form a collar around the vesicle’s neck [37]. Thus, dynamin inhibition is expected to demonstrate a coherent pattern with CME. However, the FPLM, the NP significantly inhibited by chlorpromazine, was not inhibited by dynasore. Previously, it has been shown that dynamin is not the only target of dynasore. Cholesterol, lipid rafts, and actin are identified as the “off-targets” of dynasore as well [38]. It has been reported that vesicular trafficking in MPC involves dynamic membrane changes during vesicle formation and fusion, relying on actin and cholesterol for membrane ruffle formation and cargo engulfment. Dynasore disrupts this process by lowering plasma membrane cholesterol. Therefore, it was proposed that the results observed with the dynamin application were more likely associated with inhibition of MPC than with CME (Figure 4a,e).

The mechanism of inhibition of cell internalization of nanoparticles by MβCD involves its ability to disrupt lipid rafts and cholesterol-rich membrane microdomains. MβCD interferes with caveolae and caveolae-like structures, leading to a decrease in the internalization of some toxins. This inhibition is primarily mediated through endocytosis by disrupting the cholesterol-rich microdomains and glycolipid clusters on the plasma membrane [39]. Additionally, MβCD has been shown to inhibit clathrin-independent endocytosis, which further emphasizes its role in interfering with the internalization process of specific molecules. MβCD was able to inhibit (Figure 4d) the internalization of FPCM and FPLM in a statistically meaningful manner (*p* < 0.001), while FPCM was the most inhibited one. Although MPC and CME were found to be the most effective pathways for the internalization of FPM, MβCD was not able to inhibit its endocytosis. Comparing the results obtained from the action of dynamin on the disruption of actin and cholesterol with those of MβCD in the depletion of cholesterol shows that the endocytosis of FPM by MPC is more actin-dependent. In contrast, both actin and cholesterol are involved in the endocytosis of FPCM by MPC. The endocytosis behavior of FPLM is similar to that of FPCM when using the MPC pathway.

Genistein, an inhibitor of tyrosine kinase that blocks CavME (Figure 4b), was only able to inhibit the internalization of FPCM (*p* < 0.001). Thus, CavME, which is desired in oral drug delivery to provide penetration of NPs through the intestinal membrane, became possible by surface modification of PNM with C [10].

Earlier research has associated dynamin activity with CavME; however, more recent studies have revealed that this endocytosis pathway can be activated independently of dynamin [40]. Thus, the similar patterns of inhibition of dynamin and CavME in Figure 4a,b could not be related to each other, and the ability of FPCM to use CavME as an internalization pathway is independent of its ability to use MPC, which the latter pathway is inhibited by dynasore.

Overall assessment of the results shows that the main endocytosis pathways of FPM are MPC and CME. The surface modification of FPM with C increased the number of endocytosis pathways. FPCM could enter the MCF-7 cells via all tested endocytosis pathways. Although this increased the endocytosis possibilities for FPCM, entry of this NP into the cell is not selective and targeted, as MPC is still the predominant cellular entry pathway. Coating FPM with HL reduced the ratio of MPC (*p* < 0.001) and increased the CME ratio (*p* < 0.05) compared to FPM. It could be concluded that the pathway modulation is likely due to physicochemical changes (carbohydrate–membrane interaction).

Figure 6 confirms this finding. The difference between the core size (TEM, <200 nm) and the hydrodynamic size (DLS, up to ~308 nm) is significant. This shows that FPLM and FPCM gather a lot of water molecules on the surface; for this reason, the hydrodynamic size is much larger than the actual size. This could be a reason for MPC to be the predominant endocytosis pathway of PNM [41].

In this study, quantitative uptake was evaluated by measuring FITC fluorescence in cell lysates, providing a population-averaged estimate of NDDS internalization under each condition. This bulk approach is suitable for comparing relative uptake between formulations but does not resolve cell-to-cell heterogeneity or allow rigorous gating of live versus dead cells. Future studies can therefore complement this bulk fluorescence assay with flow cytometry to validate and refine the single-cell uptake profiles.

Previous publications have also shown the internalization of PNM to immune cells, which is the major application area of MPC [42]. Based on this information, it can be concluded that the half-life of PNM will be so short unless the ratio of MPC is decreased using some special strategies. Surface modification by HL is such a strategy. It has been demonstrated that modifying the surface of PNM with lectins alters their cell internalization pathway, directing it toward CavME [43]. Also, CME was the predominant cell internalization pathway while the surface of PNM was modified with cell-penetrating peptides [44].

In this work, we hypothesized that the presence of glucose-containing (Cellulose) and fructose-containing (levan) polysaccharide shells might favor interactions with cancer cells by presenting glucose units on the surface of NDDS because tumor cells require more nutrients than healthy cells. However, it should be emphasized that the reaction between the synthesized NDDS and nutrient transporters such as Glut1 and Glut5 has not been directly demonstrated here. The shifts in the relative contributions of macropinocytosis, clathrin- and caveolae-mediated endocytosis observed for the different formulations could also arise from more general, carbohydrate–cell membrane interactions. Thus, any involvement of specific nutrient transporters must be considered hypothetical at this stage and should be addressed in future studies using receptor-blocking, knockdown, or imaging-based approaches.

A limitation of the present study is that all mechanistic experiments were performed in a single breast cancer cell line (MCF-7). As endocytic profiles differ between luminal and triple-negative breast cancers, future work will extend these investigations to additional models, including the more aggressive, hormone-independent MDA-MB-231 cell line, to verify whether the pathway shifts observed here are preserved across distinct breast cancer subtypes.

## 5. Conclusions

As a result, coating FITC-labeled PLGA NPs (FPM) with C (FPCM) and HL (FPLM) did not cause a significant change in surface charge, although the particle sizes were increased. This change in size was not significant enough to change the cellular entry pathway. While the cellular entry pathway of FPM in the MCF-7 breast cancer cell line was determined as MPC and CME, FPCM acted equally to FPM in using the MPC pathway. The entry of FPCM into the cell was enhanced across all the examined pathways, but none were as effective as MPC. FPCM was the only NP that provided CavME for PNM. FPLM provided less endocytosis via the MPC pathway, while it was more effective than FPM in the CME. In the attempts made to modify the cellular entry of PNM to date, surface modification with C and HL may stand out as a cheap and easy way, with no need for a chemical reaction. A limitation of this work is that nanoparticle uptake was quantified by bulk fluorescence in cell lysates rather than by flow cytometry; future studies will employ flow cytometry to confirm these findings at the single-cell level and to better characterize uptake heterogeneity within the cell population. The other limitation is studying the endocytosis pathway of FNM solely on the hormone-positive MCF-7 cell lines. Additional experiments need to be conducted to clarify these uncertainties. Also, future studies need to be conducted in serum-containing medium to characterize the protein corona, as well as validation of colloidal stability and uptake in other cell lines, would be necessary to more firmly support the proposed mechanisms.

## Figures and Tables

**Table 1:** The particle size distribution of PM, FPM, FPLM, and FPCM.

NP	Particle Size Distribution (Nm) by				
	Volume	Number	Intensity	PDI	Zeta Potential (mv)
PM	142.7	115.5	151.3	0.054	−3.90
FPM	270.6	137.9	285.3	0.208	−9.64
FPLM	280.6	120.8	270.4	0.182	−9.28
FPCM	308.0	209.3	292.8	0.179	−6.19

**Table 2:** The non-toxic doses of pharmacological inhibitors.

Pharmacological Inhibitor	Dynsaore	Genistein	EIPA	Chlorpromazine	Cyclodextrin
Non-toxic dose (μg/mL)	28.8	72	2.5	28.8	5000

## Data Availability

The data presented in this study are available in Supplementary Data S1 S2, and S3. Additional raw or processed data supporting the findings of this study will be provided upon reasonable request to the corresponding author.

## Supplementary Materials

The following supporting information can be downloaded at: https://www.mdpi.com/article/10.3390/pharmaceutics18010017/s1.

## Abbreviations

The following abbreviations are used in this manuscript:

PLGA	Poly (D, L-lactide-co-glycolide)
NDDS	Nanodrug delivery system
CME	Clathrin-mediated endocytosis
CavME	Caveolae-mediated endocytosis
MPC	Macropinocytosis
C	Cellulose
HL	Halomonas Levan
FPM	FITC-labeled PLGA
FPCM	FITC-labeled PLGA decorated with Cellulose
FPLM	FITC-labeled PLGA decorated with Halomonas Levan
DLS	Dynamic Light Scattering
DSC	Differential Scanning Calorimetry
