# Supplementary material for: Effects of Polysaccharide Coating on Cell-Surface Association and Endocytic Uptake of PLGA Nanomicelles in MCF-7 Cells
Source: Pharmaceutics. 2025 Dec 22;18(1):17. doi: 10.3390/pharmaceutics18010017 (PMC12845347; doi:10.3390/pharmaceutics18010017)
Supplement: Supplementary file 1 [file pharmaceutics-18-00017-s001.zip › pharmaceutics-3978258-supplementary.pdf]

Effects of Polysaccharide Coating on Cell-Surface Association and Endocytic Uptake of PLGA Nanomicelles in MCF-7 Cells

Abdulkadir Bahadir Alkan[1], Esma Nur Develi[1], Fatemeh Bahadori[*,1,2], Fahri Akbas[3], Seda Susgun[3], Zahra Eskandari[2], Ebru Toksoy Öner[4]

Supplementary Data S1. Size distribution of NPs by intensity, number and volume

PM

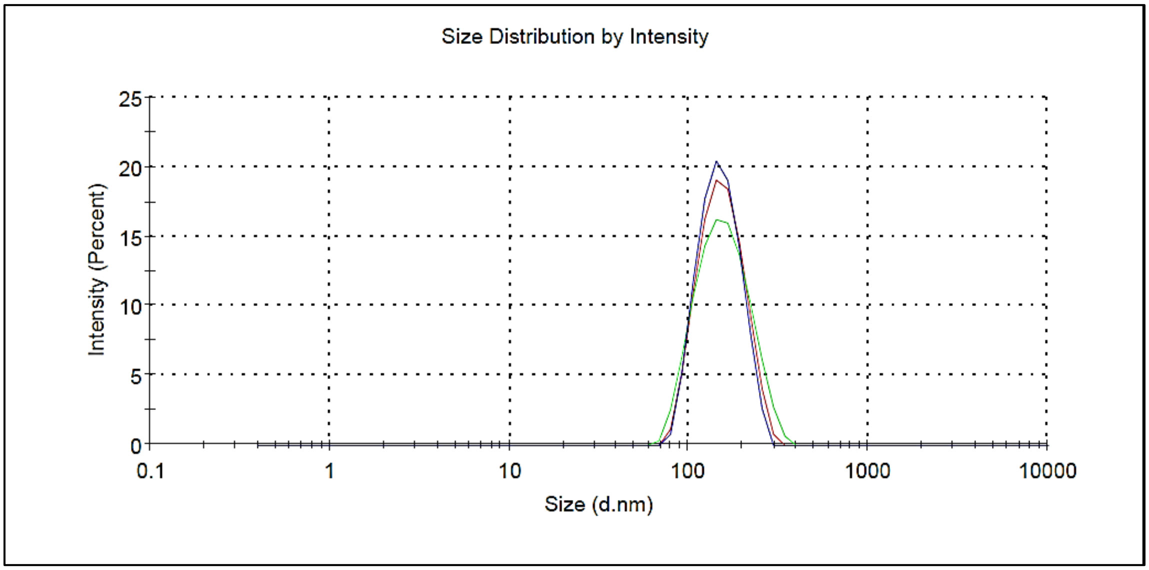

Figure S1-1: Size distribution of PM by intensity as 151.3 nm

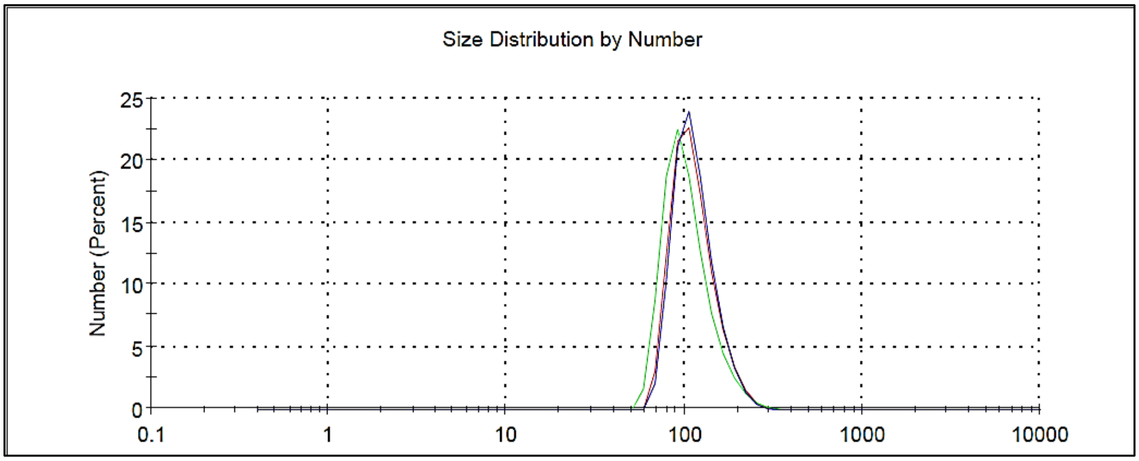

Figure S1-2: Size distribution of PM by number as 115.5 nm

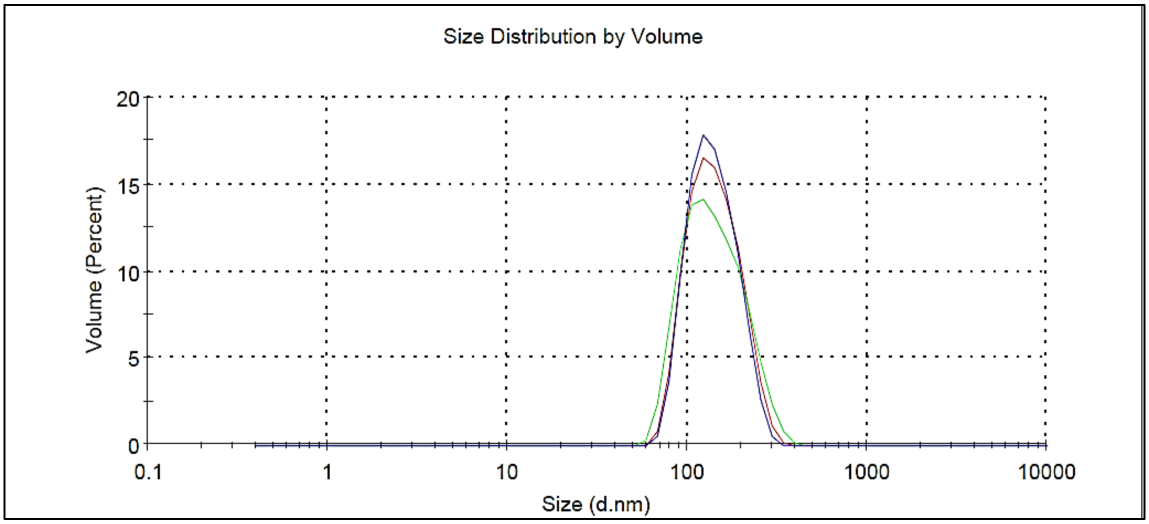

Figure S1-3: Size distribution of PM by volume as 142.7 nm

FPM

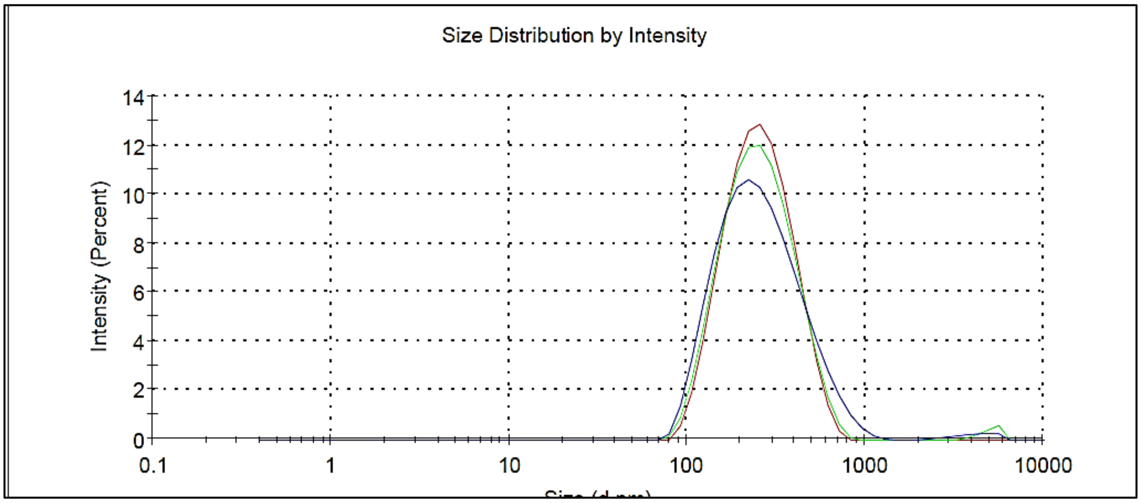

Figure S1-4: Size distribution of FPM by intensity as 285.3 nm

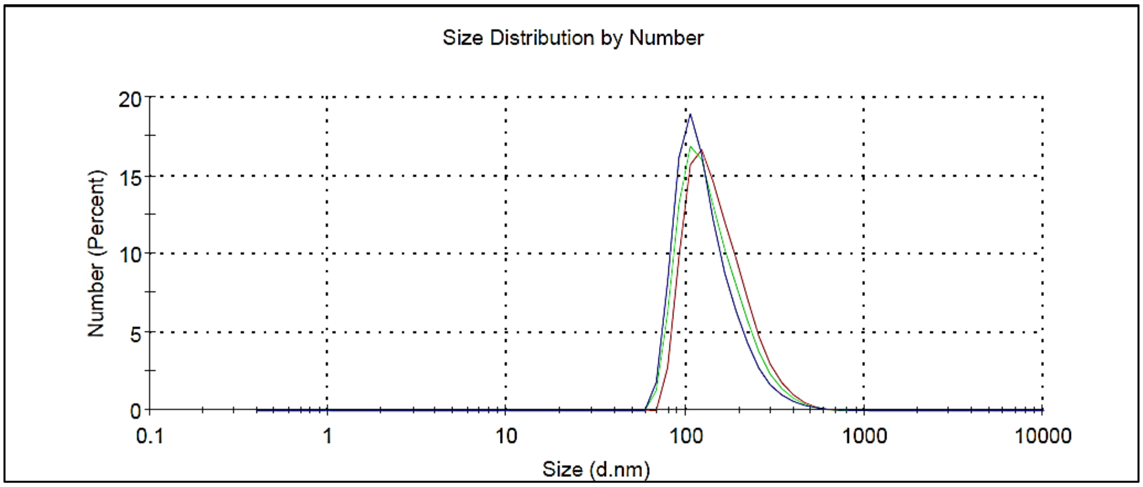

Figure S1-5: Size distribution of FPM by number as 137.9 nm

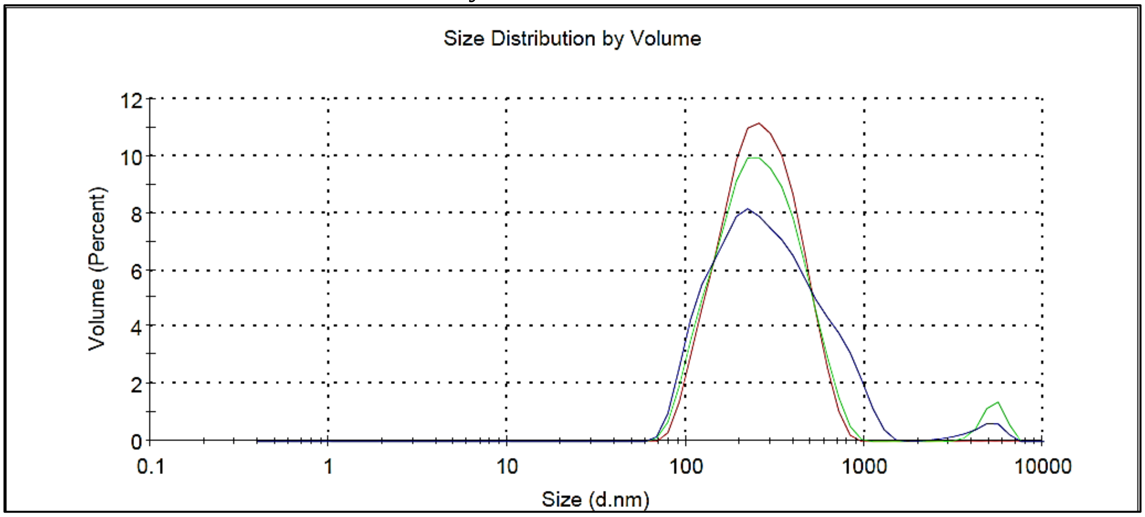

Figure S1-6: Size distribution of FPM by volume as 270.6 nm

FPLM

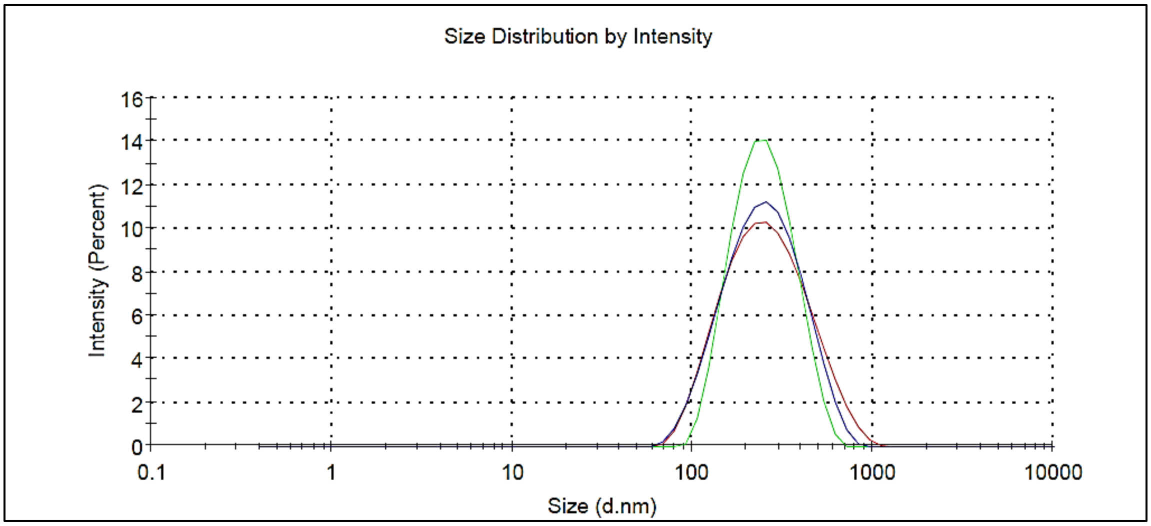

Figure S1-7: Size distribution of FPLM by intensity as 270.4 nm

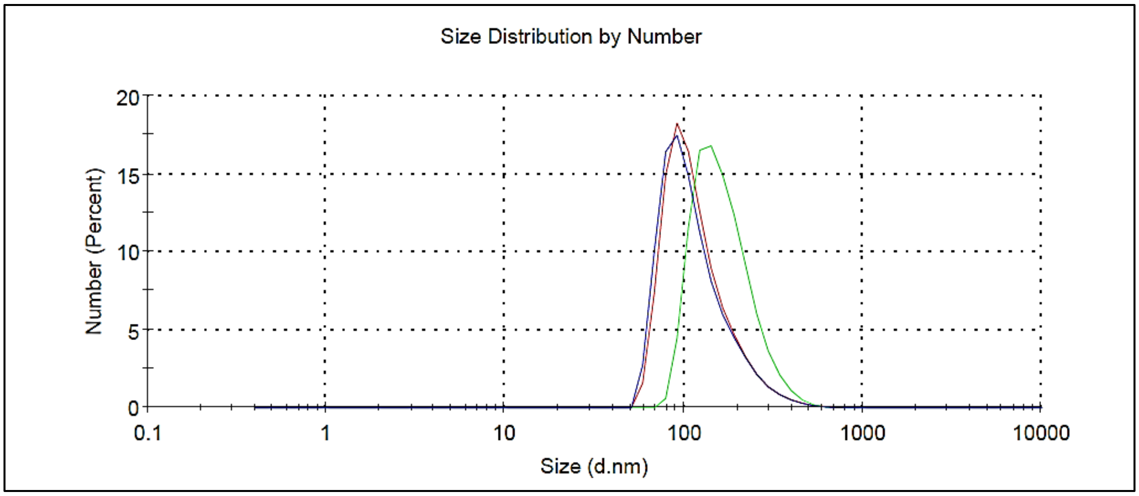

Figure S1-8: Size distribution of FPLM by number as 120.8 nm

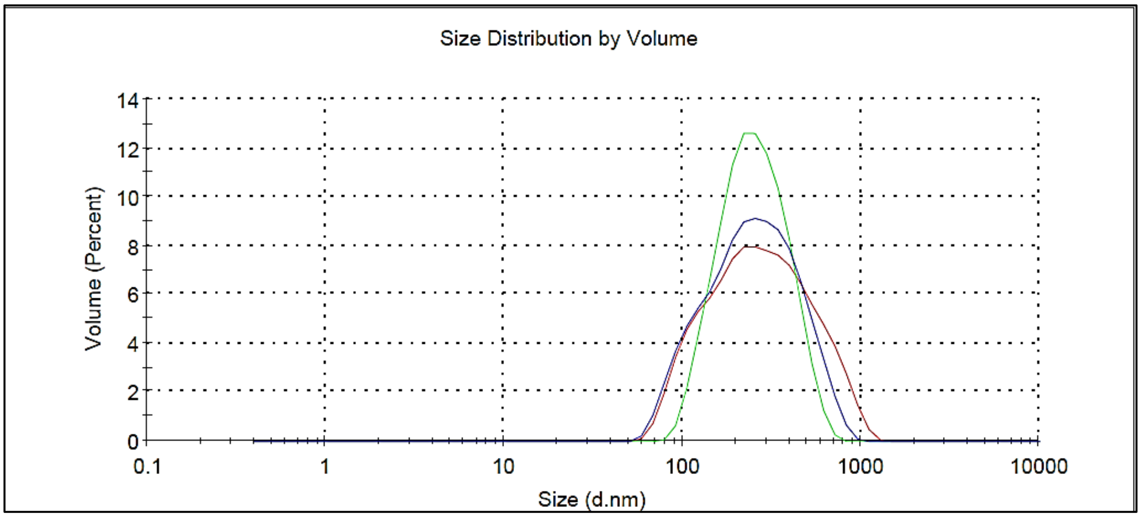

Figure S1-9: Size distribution of FPLM by volume as 280.6 nm

FPCM

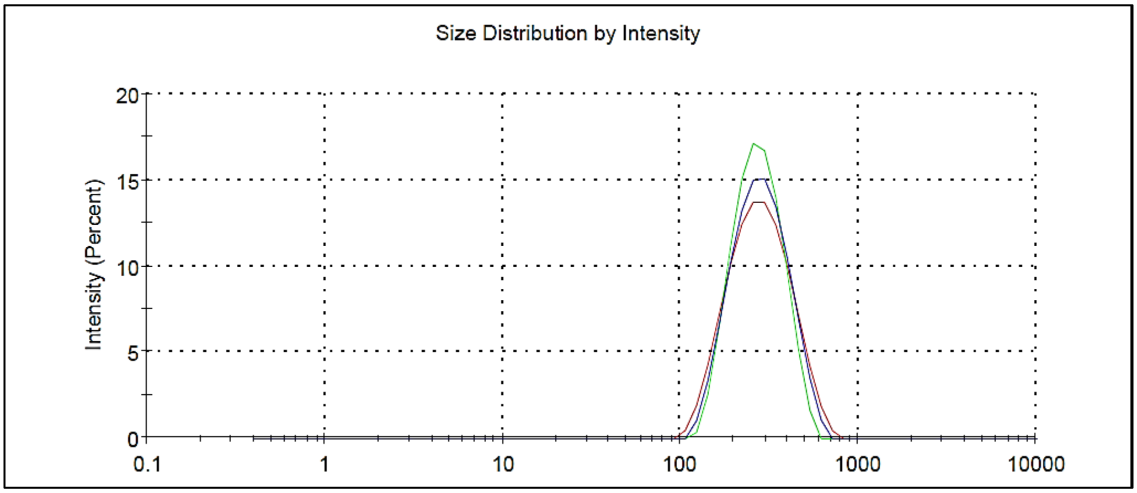

Figure S1-10: Size distribution of FPCM by intensity as 292.8 nm

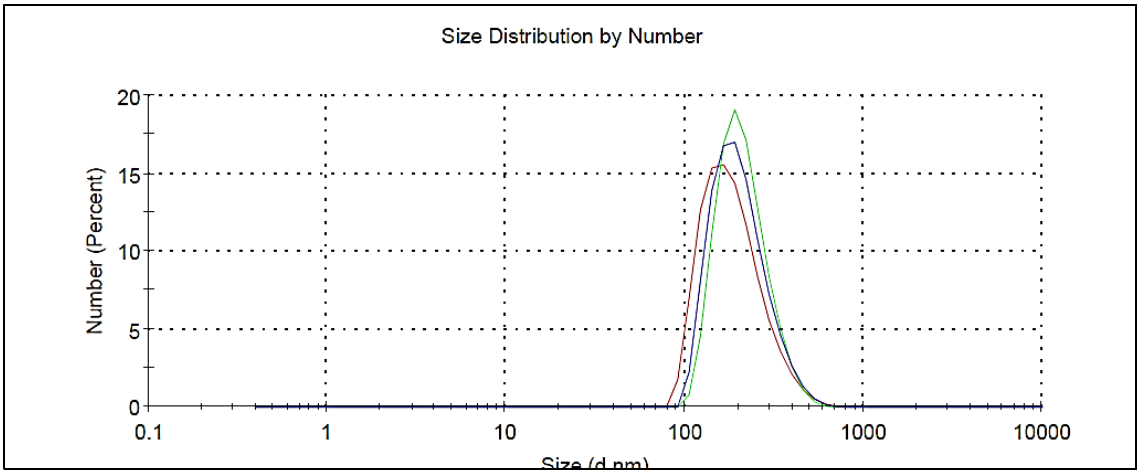

Figure S1-11: Size distribution of FPCM by number as 209.3 nm

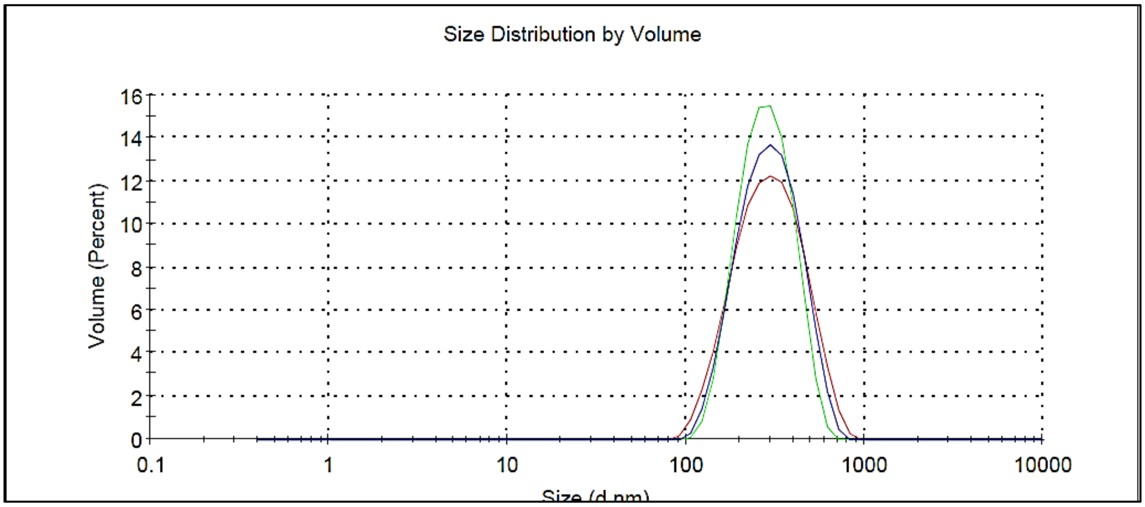

Figure S1-12: Size distribution of FPCM by volume as 308.0 nm

Zeta potential of NPs

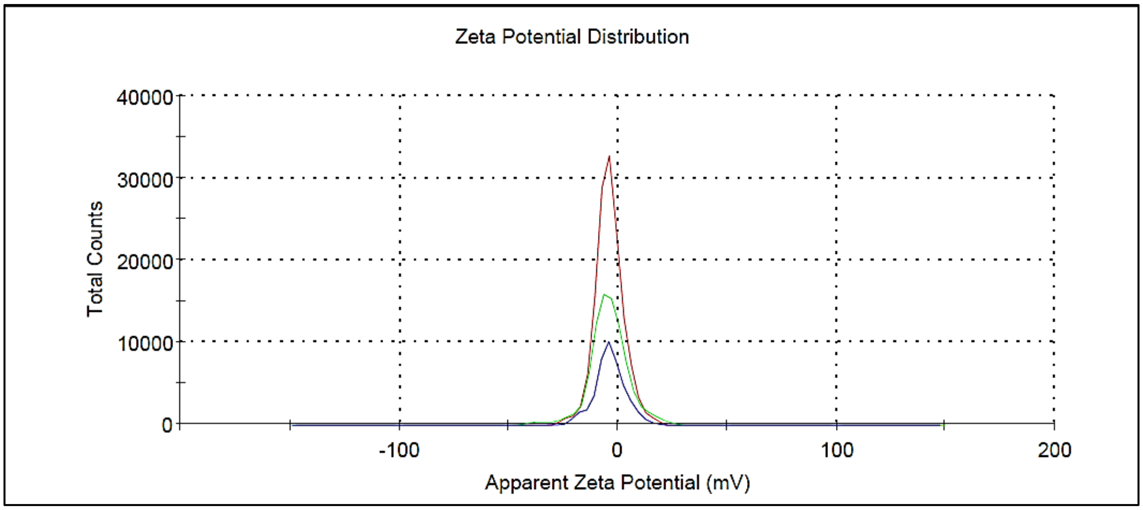

Figure S1-13: Surface zeta of PM as -3.90 mv

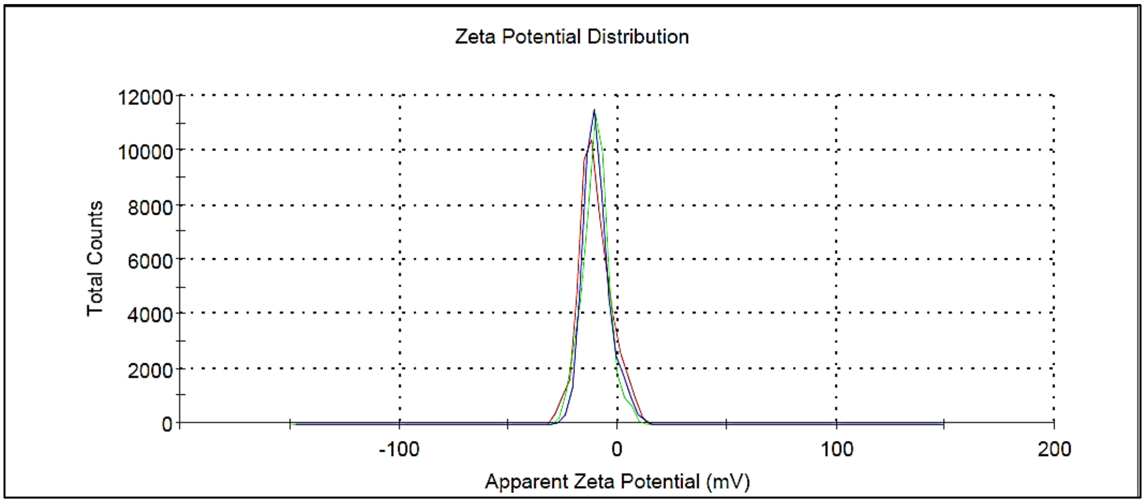

Figure S1-14: Surface zeta of FPM as -9.64 mv

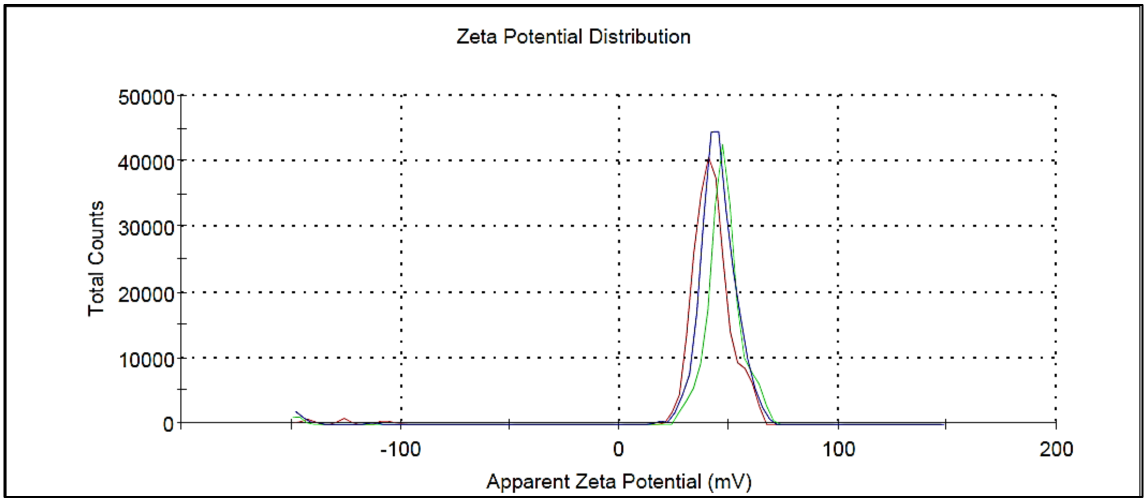

Figure S1-15: Surface zeta of FPLM as -9.28 mv

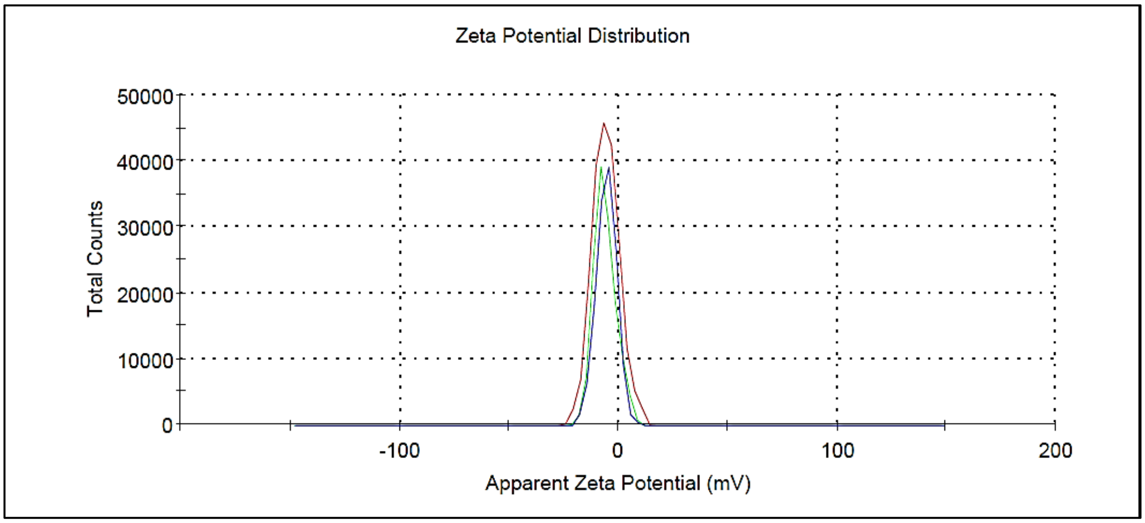

Figure S1-16: Surface zeta of FPCM as -6.19 mv

Supplementary Data 2. Fluorescent microscope images of MCF-7 cells treated with FITC-labeled PLGA NPs, a) without inhibitor, b) inhibitor; dynasore (dynamin inhibitor), c) inhibitor; chlorpromazine (clathrin inhibitor) d): genistein (caveolae inhibitor), e) inhibitor; methyl- β-cyclodextrin (cholesterol depletion agent), and f) inhibitor; EIPA, micropinocytosis inhibitor

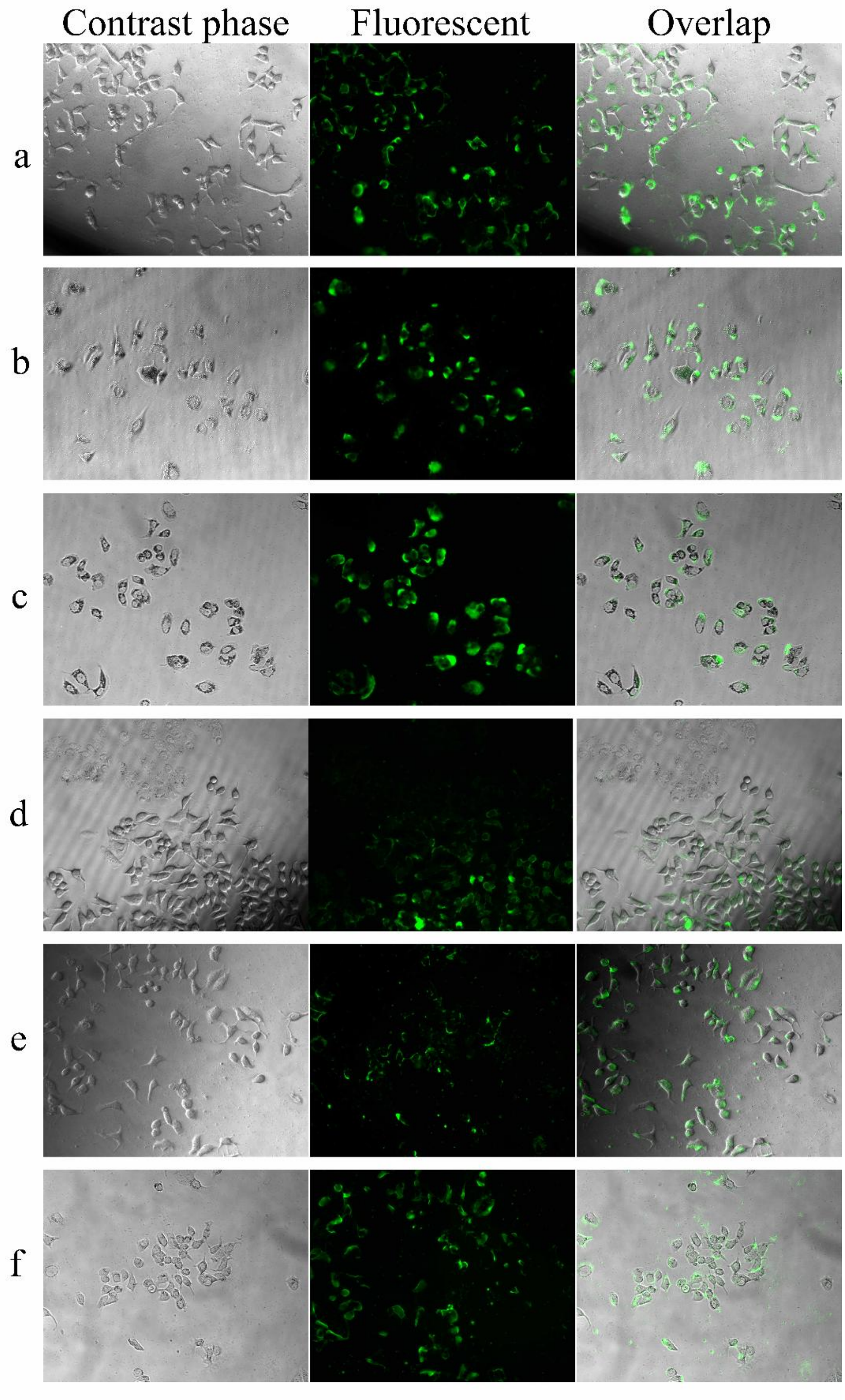

Contrast phase
Fluorescent
Overlap
a
b
c
d
e
f

Supplementary Data 3. Summary of predominant endocytosis pathways for FPM, FPCM and FPLM based on inhibitor experiments.

**Nanoparticle**	**Primary pathway (inhibitor)**	**Evidence (relative inhibition vs control, p-value)**	**Secondary pathway (inhibitor)**	**Evidence**	**Minor pathway(-s)**	**Notes**
FPM	Macropinocytosis (EIPA)	Largest significant decrease in uptake ($p < 0.001$)	CME and/or dynamin-dependent	Moderate but significant decrease ($p < 0.05$)	CavME (genistein, MβCD)	MPC-dominated uptake
FPCM	Macropinocytosis (EIPA)	Largest significant decrease in uptake ($p < 0.001$)	CME (chlorpromazine)	Second largest significant decrease ($p < 0.001$)	CavME	Similar profile to FPM
FPLM	CME (chlorpromazine)	Largest significant decrease in uptake ($p < 0.001$)	Macropinocytosis (EIPA)	Moderate significant decrease ($p < 0.001$)	CavME (genistein, MβCD)	Shift towards CME
